# Identification of *Vernonia patula* Merr. and Its Similar Varieties Based on a Combination of HPLC Fingerprinting and Chemical Pattern Recognition

**DOI:** 10.3390/molecules29071517

**Published:** 2024-03-28

**Authors:** Wen Liu, Liyuan Huang, Jiashan Zhu, Liwen Lu, Xiaoling Su, Xiaotao Hou, Zeen Xiao

**Affiliations:** Faculty of Pharmacy, Guangxi University of Chinese Medicine, Nanning 530200, China; liuw2017@gxtcmu.edu.cn (W.L.); huang6882024@163.com (L.H.); 13877241685@163.com (J.Z.); 18776519324@163.com (L.L.); s18778695492@163.com (X.S.)

**Keywords:** *Vernonia patula* Merr., high-performance liquid chromatography, similar varieties, identification, chemometrics

## Abstract

*Vernonia patula* Merr. (VP) is a traditional medicine used by the Zhuang and Yao people, known for its therapeutic properties in treating anemopyretic cold and other diseases. Distinguishing VP from similar varieties such as *Praxelis clematidea* (PC), *Ageratum conyzoides* L. (AC) and *Ageratum houstonianum* Mill (AH) was challenging due to their similar traits and plant morphology. The HPLC fingerprints of 40 batches of VP and three similar varieties were established. SPSS 20.0 and SIMCA-P 13.0 were used to statistically analyze the chromatographic peak areas of 37 components. The results showed that the similarity of the HPLC fingerprints for each of the four varieties was >0.9, while the similarity between the control chromatogram of VP and its similar varieties was <0.678. Cluster analysis and partial least squares discriminant analysis provided consistent results, indicating that all four varieties could be individually clustered together. Through further analysis, we found isochlorogenic acid A and isochlorogenic acid C were present only in the original VP, while preconene II was present in the three similar varieties of VP. These three components are expected to be identification points for accurately distinguishing VP from PC, AC and AH.

## 1. Introduction

**Figure 1:**
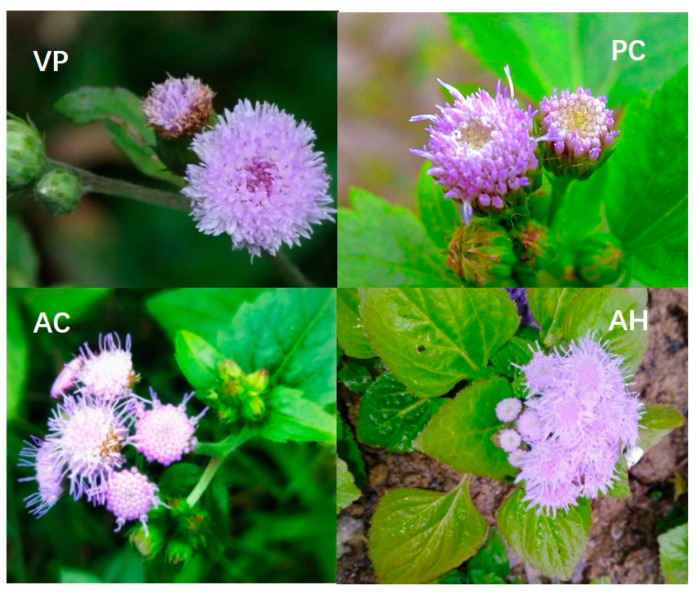
Plant morphology diagram of VP, PC, AC and AH.

*Vernonia patula* Merr. (VP) is primarily distributed in South China [1]. It has been found to have multiple therapeutic effects, including removing pathogenic heat from the blood and toxic material from the body, as well as clearing heat and promoting diuresis. Furthermore, it is used in the treatment of various conditions such as anemopyretic cold, headaches, hypertension, mastitis, malaria, heat leak, eruptive disease, eczema and urticaria [2]. Extensive research has been conducted by both domestic and international researchers on the chemical composition of this genus. It has been discovered that the main components of VP include sesquiterpenoids, triterpenoids, flavonoids, steroids and volatile oils [3,4,5]. These components exhibit diverse pharmacological activities, including antitumor, antifungal and antimalarial effects [5,6,7]. PC, AC and AH [8,9] are all in the Asteraceae family, belonging to invasive plants. PC belongs to the *Zeeland* genus, while AC and AH belong to the *Agastache* genus. The plant morphology of three invasive species is very similar to that of VP [10], as shown in Figure 1. Our group has previously conducted quality control research on VP [11,12]. Due to their similar traits and plant morphology, it is easy to confuse VP with PC, AC and AH. Therefore, establishing an analytical method to distinguish VP and its similar varieties is of great significance. There have been no reports about it. Our research aims to utilize the HPLC method [13,14] for accurate differentiation of VP and three similar varieties, ensuring their safe and effective clinical use while preventing mis-harvest and mis-collection. Fingerprints of VP and its three similar varieties have been established, and further analysis was carried out through cluster analysis [15,16,17,18] and partial least squares discriminant analysis [16,17,19] to identify the distinguishing points among these varieties.

## 2. Results

### 2.1. Validation of Fingerprint Analysis Method

#### 2.1.1. The Results of Precision

As shown in Table 1 and Table 2, the results showed that the relative standard deviation (RSD) of the relative retention time and relative peak area was less than 0.23% and 2.8%, respectively, which indicated good precision of the instrument and meeting the requirements of HPLC determination.

#### 2.1.2. The Results of Stability Test

As shown in Table 3 and Table 4. The results showed that the RSD of relative retention time and relative peak area were less than 0.19% and 2.8%, respectively, which indicated that the VP test solution had good stability when stored at room temperature for 24 h and met the requirements for HPLC determination.

#### 2.1.3. The Results of Repeatability Test

As shown in Table 5 and Table 6. The results showed that the RSD of relative retention time and relative peak area were less than 0.10% and 2.9%, respectively, which indicated that the method had good repeatability and met the requirements of HPLC determination.

### 2.2. Establishment of HPLC Fingerprints

#### 2.2.1. Identification of Common Peaks

**Figure 2:**
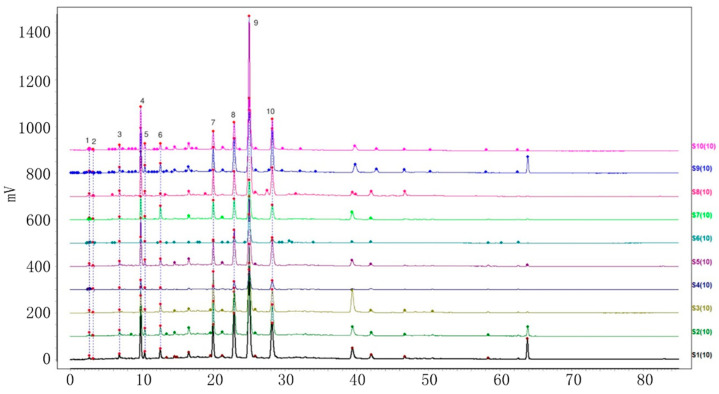
Chromatogram of ten batches of VP 10 shared peaks superimposed.

**Figure 3:**
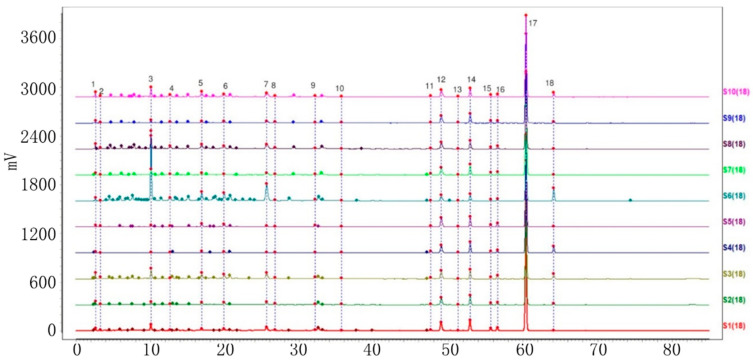
Chromatogram of ten batches of PC with 18 shared peaks superimposed.

**Figure 4:**
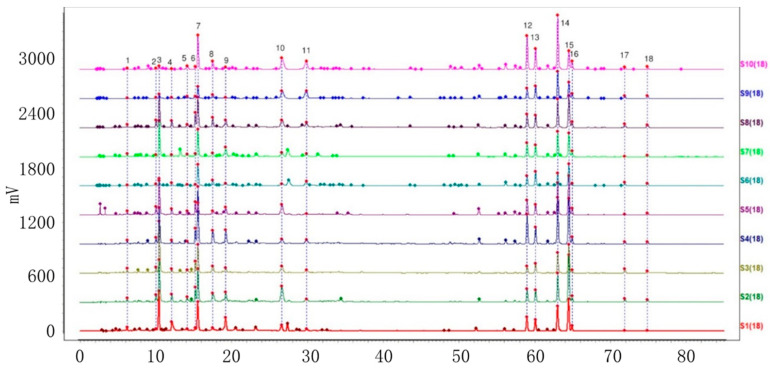
Chromatogram of ten batches of AC with 18 shared peaks superimposed.

**Figure 5:**
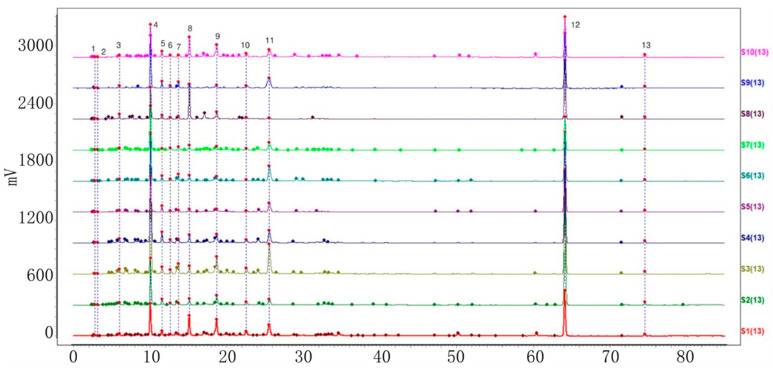
Chromatogram of ten batches of AH with 13 shared peaks superimposed.

Ten batches of powdered VP, PC, AC, and AH were collected and prepared. The sample solutions were injected into the HPLC system, following the method demonstrated under ‘Item 4.3’. Further analysis was carried out using the specified chromatographic conditions outlined in ‘Item 4.3’. The HPLC fingerprints of the four varieties with ten batches each were acquired and subsequently imported into the chromatographic similarity assessment system ‘Similarity Evaluation System for Chromatographic Fingerprints of Traditional Chinese Medicine’ (V 2012). The first batch of each variety was designated as the reference fingerprint. The median method was utilized, with a time window width of 0.5. Peaks with a separation greater than 1.5 were calibrated at multiple points and automatically matched to generate the superimposed chromatograms of ten batches of each of the four objective varieties. Furthermore, the characteristic peaks were sequentially tagged with consecutive numbers (1, 2, 3, …, N), and a total of 10, 18, 18, and 13 characteristic peaks were labeled (Figure 2, Figure 3, Figure 4 and Figure 5). The cumulative area of these characteristic peaks in each variety accounted for more than 92% of the total peak areas, thus confirming them as common peaks in the HPLC fingerprints of these four varieties.

**Figure 6:**
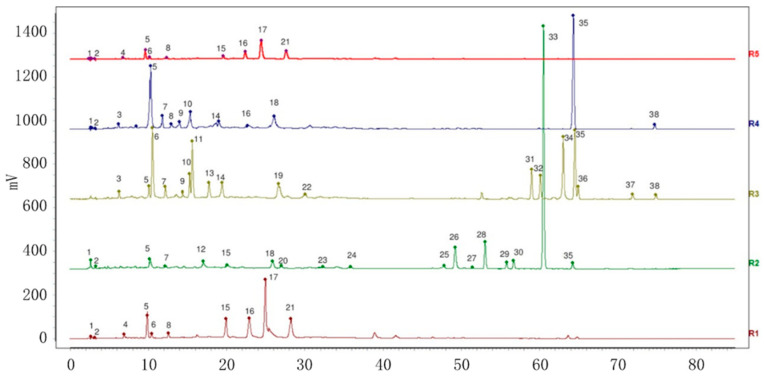
HPLC control map of VP (R1), PC (R2), AC (R3), AH (R4) and mixed control (R5). (Numbers of 1–38 were the common peaks of VP (R1), PC (R2), AC (R3) and AH (R4); 4 Neochlorogenic acid; 5 Chlorogenic acid; 6 Cryptochlorogenic acid; 8 Caffeic acid; 15 Isoquercitrin; 16 Isochlorogenic acid B; 17 Isochlorogenic acid A; 21 Isochlorogenic acid C).

The retention times of the peaks shared by the four varieties were compared to those of the control. After renumbering, eight chromatographic peaks were identified as neochlorogenic acid (peak 4), chlorogenic acid (peak 5), cryptochlorogenic acid (peak 6), caffeic acid (peak 8), isoquercitrin (peak 15), isochlorogenic acid B (peak 16), isochlorogenic acid A (peak 17) and isochlorogenic acid C (peak 21). Figure 6 shows the reference fingerprints (R) of the four varieties and the superimposed diagram of the mixed standard samples.

#### 2.2.2. Similarity Analysis of HPLC Fingerprints of Individual Variety

The similarity analysis of HPLC fingerprints of VP and three similar varieties was conducted using the Similarity Evaluation System for Chromatographic Fingerprints of Traditional Chinese Medicine (V 2012). The results indicated that the similarity values of the four varieties (ten batches each) were greater than 0.9, demonstrating a high degree of similarity among the fingerprints of all varieties.

#### 2.2.3. HPLC Fingerprint Similarity Analysis of VP and Three Similar Varieties

The chromatographic data of VP (the reference fingerprint R) and PC (S1–S10), AC (S1–S10) and AH (S1–S10) were imported into the Similarity Evaluation System for Chromatographic Fingerprints of Traditional Chinese Medicine (V 2012). The results revealed that the similarity between VP and PC, AC and AH were <0.13, <0.49 and <0.68, respectively. These results indicated significant differences in the chemical composition between VP and three similar varieties.

### 2.3. Chemical Pattern Recognition

#### 2.3.1. Cluster Analysis (CA)

**Figure 7:**
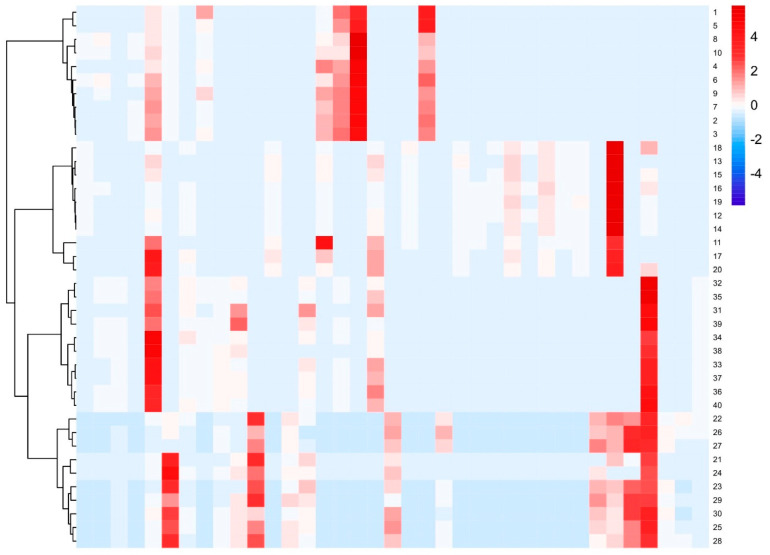
Heatmap and cluster analysis of 40 batches of VP and its similar varieties (1–10 VP; 11–20 PC; 21–30 AC; 31–40 AH).

The common peak data obtained from the fingerprint spectrum were imported into the analysis website (https://hiplot.com.cn/ accessed on 10 October 2023) for heat map and CA (Figure 7). The CA of the heat map revealed that 40 samples could be clustered into two categories. One category consisted of 10 batches of VP, while the other category consisted of 30 batches of three similar varieties. This distinction successfully demonstrated that the chemical composition of VP differed from that of the other three similar varieties.

#### 2.3.2. Partial Least Squares Discriminant Analysis (PLS-DA)

**Figure 8:**
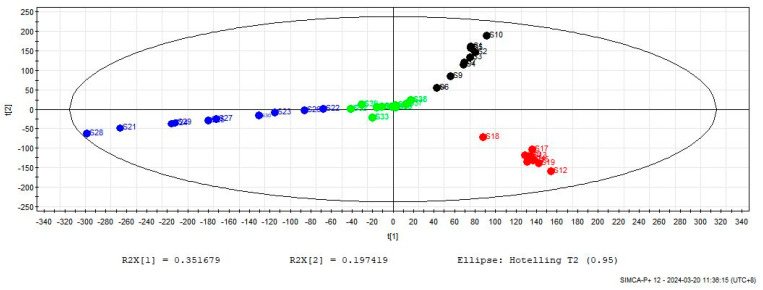
PLS-DA score plot for 40 batches of VP and its similar varieties. (1–10 VP (Black); 11–20 PC (Red); 21–30 AC (Blue); 31–40 AH (Green)).

**Figure 9:**
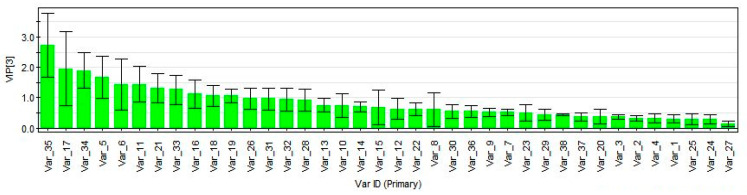
VIP of 38 chromatographic peak variables for 40 batches of VP and its similar varieties.

The chromatographic peak areas of 38 components from 40 batches of four varieties were imported into SIMCA-P 12.0 for analysis. Each variety was assigned as a class and subjected to PLS-DA analysis. The two-dimensional score diagram in Figure 8 showed that all ten batches of each variety clustered together without any overlap. Additionally, VP was found to be significantly distinct from three similar varieties, which aligns with the results of CA. To further investigate, VP was grouped as one class and the other three as another class, and a PLS-DA analysis was conducted. Figure 9 presents the projection importance index (VIP) of the 38 peaks. The results revealed that the VIP values of peaks 35, 17 (isochlorogenic acid A), 34, 5 (chlorogenic acid), 6 (cryptochlorogenic acid), 11, 21 (isochlorogenic acid C), 33, 16 (isochlorogenic acid B), 18 and 19 were all greater than 1, indicating that these substances were the key components responsible for the main differences.

When combined with the original spectrum, it is observed that isochlorogenic acids A and isochlorogenic acids C were exclusively present in the genuine VP, while peak 35 (unknown) was only found in three similar varieties. Therefore, these three substances can serve as markers to authenticate the VP.

#### 2.3.3. Separation and Identification of Peak 35

The separation of peak 34 was performed using the preparative liquid chromatography technique. The chromatographic column used was YMC-Pack ODS-A (5 µm, 12 nm, YMC, Tokyo, Japan), with dimensions of 250 × 20 mm. The mobile phase consisted of acetonitrile water (55:45), and the flow rate was set at 4 mL/min. The detection wavelength used was 327 nm. The separation sample was AH. The structure was elucidated through the analysis of NMR and MS data.

**Figure 10:**
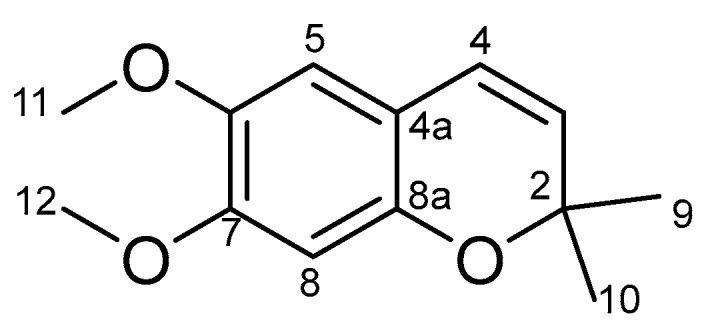
The structure of preconene II.

Preconene II (Figure 10): yellow powder. ^1^H NMR (500 MHz, CDCl_3_, *δ*, ppm, *J*/Hz): 6.52 (1H, s, H-8), 6.40 (1H, s, H-5), 6.22 (1H, d, *J* = 9.7, H-4), 5.46 (1H, d, *J* = 9.7, H-3), 3.82 (3H, s, H-11), 3.81 (3H, s, H-12), 1.40 (6H, s, H-9/10) (see Supplementary Materials Figure S1). ^13^C NMR (125 MHz, CDCl_3_, *δ*, ppm): 149.7 (C-7), 147.3 (C-8a), 143.2 (C-6), 128.3 (C-3), 122.1 (C-4), 113.1 (C-4a), 109.8 (C-5), 101.1 (C-8), 76.1 (C-2), 56.6 (C-11), 56.0 (C-12), 27.8 (C-9/10) (see Supplementary Materials Figure S2).

## 3. Discussion

To establish an attractive and informative peak shape of the fingerprints, the impact of various extraction solutions (ethanol, methanol, 90% methanol, 75% methanol, 90% ethanol and 75% ethanol) on the peak area and shape were evaluated. The results showed that 75% methanol was the most efficient extraction solution. By comparing different wavelengths through full wavelength scanning, 327 nm was found to be the optimal detection wavelength. Furthermore, the column temperature, flow rate and mobile phase composition were examined. The column temperature was finally determined as 35 °C, flow rate as 1 mL/min, injection volume as 10 µL, and the mobile phase as a 0.2% phosphoric acid aqueous solution-acetonitrile. The gradient elution procedure was described in Section 2.3. The HPLC fingerprints revealed that the similarity of the four varieties was >0.9, indicating a good similarity in the fingerprints of each variety. Furthermore, the similarity between VP and PC, AC, and AH was less than 0.13, 0.49 and 0.68, respectively, indicating that these substances had relatively large differences between VP and three similar varieties. Since isochlorogenic acid A and isochlorogenic acid C were found only in the genuine VP, while preconene II was only detected in the other three varieties, these three substances can be used as identification points to verify the authenticity of VP.

## 4. Materials and Methods

### 4.1. Plant Materials, Chemicals and Reagents

Various batches of VP, PC, AC and AH were obtained from Guangxi, China, and were identified by Associate Professor Dai Zhonghua of the Department of Medicinal Plants, Guangxi University of Chinese Medicine. Reference substances, including neochlorogenic acid (C_16_H_18_O_9_, RP211214), chlorogenic acid (C_16_H_18_O_9_, RP210505), cryptochlorogenic acid (C_16_H_18_O_9_, RP200726), caffeic acid (C_9_H_8_O_4_, RP220610), isoquercitrin (C_21_H_20_O_12_, RP200404), isochlorogenic acids A (C_25_H_24_O_12_, RP220616), sochlorogenic acids B (C_25_H_24_O_12_, RP220619) and sochlorogenic acids C (C_25_H_24_O_12_, RP201026) were all purchased from Chengdu Maidesheng Technology Co., Ltd., Chengdu, China with a purity of above 98%. CDCl_3_ was purchased from Sigma Corporation (Cream Ridge, NJ, USA), and HPLC grade Acetonitrile (F22M6E202) was purchased from Thermo Fisher Scientific Co. (Waltham, MA, USA). Analytical grade methanol was purchased from Chengdu Kelong Chemical Co., Ltd. (Chengdu, China); phosphoric acid was purchased from Sinopharm Chemical Reagent Co., Ltd. (Shanghai, China).

### 4.2. Apparatus

The Agilent 1100 HPLC system (Agilent Technologies, Santa Clara, CA, USA) was used, which consisted of a G1311A four-way pump, G1322A degasser, G1313A automatic sampler, G1315B diode array detector, and G1316A column temperature chamber; ME204/02 electronic balance (Mettler Toledo Instruments (Shanghai) Co., Ltd., Shanghai, China); circulating water multi-purpose vacuum pump (UPC-II-10T, Zhengzhou Greatwall Scientific Industrial and Trade Co., Ltd., Zhengzhou, China); ultrasonic cleaner (KQ5200B, Kun Shan Ultrasonic Instruments Co., Kunshan, China); pure water instrument (UPC-II-10T, Sichuan ULUPURE Ultrapure Technology Co., Ltd., Chengdu, China); Bruker Avance 500M nuclear magnetic resonance instrument (Bruker, Fällanden, Switzerland); LC-20AR Shimadzu preparative liquid chromatograph (Shimadzu, Kyoto, Japan).

### 4.3. Fingerprints of VP and Three Similar Varieties

To prepare the test solution, follow these steps. First, weigh 2.5 g of VP, PC, AC and AH powder individually and accurately. Place each powder in a 100 mL conical flask with a stopper. Next, add 25 mL of 75% methanol accurately. Record the weight of the flask and its contents. Then, extract the mixture using ultrasound for 30 min. Allow the flask to cool and weigh it again to determine the weight loss. Fill the flask with 75% methanol to compensate for the weight loss. Shake the mixture well and let it stand. Finally, filter the supernatant using a microporous membrane with a pore size of 0.22 μm. Collect the continuous filtrate to obtain the test solution. The neochlorogenic acid, chlorogenic acid, cryptochlorogenic acid, caffeic acid, isoquercitrin, isochlorogenic acid B, isochlorogenic acid A and isochlorogenic acid C were accurately weighed and dissolved in 75% methanol to obtain a mixed standard solution. The concentrations of the above standard solutions were 25, 125, 25, 15, 200, 250, 500 and 375 μg/mL, respectively. The standard solutions were stored in a refrigerator at 4 °C for future use.

The analysis was conducted using a Zafex Supfer HP-C_18_ column (4.6 mm × 250 mm, 5 µm) with a mobile phase consisting of a 0.2% phosphoric acid aqueous solution and acetonitrile. A 10 µL injection volume was used, and the column temperature was maintained at 35 °C and a flow rate of 1 mL/min. The detection wavelength was set at 327 nm. The gradient elution conditions are shown in Table 7.

### 4.4. Validation of HPLC Methodology

#### 4.4.1. Precision Experiment

Take 2.5 g of VP powder (S10), accurately weigh it, prepare the VP test solution according to the preparation method of the test substance under Section 4.3, and inject it continuously six times according to the chromatographic conditions under Section 4.3. Additionally, peak 17 (isochlorogenic acid A) was selected as the reference peak due to its consistent presence in all VP samples tested, as well as its high peak height and good separation. Subsequently, the RSD of relative retention time and relative peak area were calculated for all common peaks.

#### 4.4.2. Stability Test

Take six portions of VP powder (S10), each weighing 2.5 g, accurately weigh, and prepare the VP test solution according to the preparation method of the test substance under Section 4.3. Conduct sample studies at 0 h, 2 h, 4 h, 8 h, 12 h, and 24 h according to the chromatographic conditions under Section 4.3. Additionally, peak 17 (isochlorogenic acid A) was selected as the reference peak due to its consistent presence in all VP samples tested, as well as its high peak height and good separation. Subsequently, the RSD of relative retention time and relative peak area was calculated for all common peaks.

#### 4.4.3. Repeatability Test

Take six portions of VP powder (S10), each weighing 2.5 g, accurately weigh, and prepare the VP test solution according to the preparation method of the test substance in Section 4.3. Inject continuously according to the chromatographic conditions in Section 4.3. Additionally, peak 17 (isochlorogenic acid A) was selected as the reference peak due to its consistent presence in all VP samples tested, as well as its high peak height and good separation. Subsequently, the RSD of relative retention time and relative peak area was calculated for all common peaks.

### 4.5. Data Analysis

The peak calibration and similarity analysis were conducted using the software ‘Chromatographic fingerprint similarity evaluation system of traditional Chinese medicine’ (V 2012), which was recommended by the China Pharmacopoeia Commission. The similarity was measured using the vector angle cosine technique. Additionally, CA and PLS-DA were performed on the chromatographic peak areas of 37 components of four varieties using SPSS (SPSS USA, v20.0) and SIMCA-P 12.0.

## 5. Conclusions

The HPLC fingerprints of VP and three similar varieties (ten batches each) were established using the HPLC method. A similarity analysis was conducted which revealed a similarity of less than 0.678 between the control map of VP and the other three varieties. This suggested that the chemical composition of VP and its similar varieties were different, and the developed fingerprints can aid in distinguishing between the genuine VP and the counterfeit. The CA and PLS-DA methods were used to analyze the chromatographic peak areas of 38 components in 40 batches of VP and three similar varieties as data samples. The results showed that ten batches of each variety could be clustered together. The VIP values of 35 (preconene II), 17 (isochlorogenic acid A), 34, 5 (chlorogenic acid), 6 (cryptochlorogenic acid), 11, 21 (isochlorogenic acid C), 33, 16 (isochlorogenic acid B), 18 and 19 were all greater than 1, indicating significant differences between VP and its similar varieties. Among them, isochlorogenic acid A, isochlorogenic acid C and preconene II could be used as identification points to determine the authenticity of VP, meaning that the genuine VP should always include both isochlorogenic acids A and isochlorogenic acids C, while preconene II should not be detected.

## Figures and Tables

**Table 1:** Relative retention time of precision test.

No.	1	2	3	4	5	6	RSD%
Peak 1	0.1044	0.1041	0.1040	0.1041	0.1041	0.1041	0.12
Peak 2	0.2469	0.2455	0.2453	0.2455	0.2454	0.2454	0.23
Peak 4	0.3491	0.3483	0.3483	0.3483	0.3483	0.3480	0.11
Peak 5	0.3713	0.3704	0.3705	0.3706	0.3706	0.3703	0.090
Peak 6	0.4500	0.4494	0.4495	0.4496	0.4496	0.4493	0.050
Peak 8	0.6325	0.6322	0.6319	0.6321	0.6320	0.6320	0.030
Peak 15	0.8117	0.8118	0.8118	0.8120	0.8118	0.8118	0.010
Peak 16	0.8848	0.8848	0.8848	0.8848	0.8848	0.8848	0.00
Peak 17	1.0000	1.0000	1.0000	1.0000	1.0000	1.0000	0.020
Peak 21	1.4148	1.4139	1.4135	1.4139	1.4135	1.4133	0.030

**Table 2:** Relative Peak Area of Precision Test.

No.	1	2	3	4	5	6	RSD%
Peak 1	0.0066	0.0062	0.0062	0.0062	0.0062	0.0062	2.6
Peak 2	0.0503	0.0512	0.0513	0.0513	0.0512	0.0513	0.71
Peak 4	0.0207	0.0206	0.0199	0.0219	0.0208	0.0208	2.8
Peak 5	0.2156	0.2163	0.2160	0.2167	0.2162	0.2164	0.15
Peak 6	0.0675	0.0676	0.0676	0.0678	0.0677	0.0679	0.17
Peak 8	0.0463	0.0462	0.0465	0.0467	0.0469	0.0466	0.51
Peak 15	0.0410	0.0383	0.0387	0.0392	0.0388	0.0408	2.6
Peak 16	0.1962	0.1962	0.1968	0.1969	0.1968	0.1970	0.17
Peak 17	1.0000	1.0000	1.0000	1.0000	1.0000	1.0000	0.00
Peak 21	0.2468	0.2464	0.2465	0.2471	0.2476	0.2475	0.19

**Table 3:** Relative retention time of stability test.

No.	1	2	3	4	5	6	RSD%
Peak 1	0.1091	0.1090	0.1091	0.1090	0.1087	0.1087	0.17
Peak 2	0.1202	0.1201	0.1201	0.1201	0.1198	0.1198	0.12
Peak 4	0.2773	0.2770	0.2773	0.2770	0.2771	0.2769	0.060
Peak 5	0.3931	0.3926	0.3928	0.3929	0.3933	0.3928	0.050
Peak 6	0.4179	0.4175	0.4177	0.4177	0.4182	0.4180	0.060
Peak 8	0.5118	0.5117	0.5116	0.5117	0.5098	0.5095	0.19
Peak 15	0.8061	0.8066	0.8065	0.8064	0.8029	0.8037	0.18
Peak 16	0.9177	0.9179	0.9179	0.9177	0.9175	0.9177	0.010
Peak 17	1.0000	1.0000	1.0000	1.0000	1.0000	1.0000	0.00
Peak 21	1.1269	1.1269	1.1273	1.1270	1.1282	1.1277	0.040

**Table 4:** Relative Peak Area of Stability Test.

No.	1	2	3	4	5	6	RSD%
Peak 1	0.0270	0.0265	0.0269	0.0267	0.0238	0.0235	0.77
Peak 2	0.0448	0.0440	0.0445	0.0443	0.0470	0.0462	0.80
Peak 4	0.0250	0.0243	0.0248	0.0252	0.0174	0.0188	2.1
Peak 5	0.1740	0.1725	0.1744	0.1736	0.1706	0.1685	0.90
Peak 6	0.0215	0.0227	0.0257	0.0295	0.0313	0.0331	2.8
Peak 8	0.0303	0.0333	0.0316	0.0322	0.0332	0.0357	0.45
Peak 15	0.0436	0.0437	0.0437	0.0435	0.0461	0.0484	1.1
Peak 16	0.1979	0.1949	0.1977	0.1954	0.1910	0.1882	0.34
Peak 17	1.0000	1.0000	1.0000	1.0000	1.0000	1.0000	0.00
Peak 21	0.2869	0.2779	0.2781	0.2804	0.2631	0.2564	2.6

**Table 5:** Relative retention time of repeatability tests.

No.	1	2	3	4	5	6	RSD%
Peak1	0.1018	0.1018	0.1017	0.1019	0.1019	0.1017	0.070
Peak 2	0.1176	0.1176	0.1176	0.1177	0.1177	0.1174	0.080
Peak 4	0.2778	0.2782	0.2774	0.2774	0.2778	0.2775	0.10
Peak 5	0.3952	0.3959	0.3952	0.3947	0.3950	0.3950	0.090
Peak 6	0.4191	0.4196	0.4190	0.4185	0.4185	0.4189	0.090
Peak 8	0.5066	0.5050	0.5062	0.5060	0.5064	0.5065	0.10
Peak 15	0.8030	0.8035	0.8042	0.8037	0.8040	0.8029	0.060
Peak 16	0.9178	0.9174	0.9184	0.9179	0.9180	0.9179	0.030
Peak 17	1.000	1.000	1.000	1.000	1.000	1.000	0.00
Peak 21	1.132	1.132	1.133	1.133	1.133	1.132	0.060

**Table 6:** Relative Peak Area of Repeatability Test.

No.	1	2	3	4	5	6	RSD%
Peak1	0.0077	0.0077	0.0076	0.0076	0.0077	0.0076	0.70
Peak 2	0.0519	0.0519	0.0517	0.0517	0.0524	0.0526	0.70
Peak 4	0.0141	0.0145	0.0142	0.0145	0.0144	0.0150	2.0
Peak 5	0.1783	0.1796	0.1805	0.1808	0.1833	0.1873	1.6
Peak 6	0.0227	0.0239	0.0247	0.0242	0.0232	0.0240	2.7
Peak 8	0.0331	0.0332	0.0328	0.0330	0.0339	0.0333	1.0
Peak 15	0.0503	0.0505	0.0516	0.0512	0.0519	0.0515	1.1
Peak 16	0.1833	0.1834	0.1852	0.1836	0.1864	0.1827	0.70
Peak 17	1.0000	1.0000	1.0000	1.0000	1.0000	1.0000	0.00
Peak 21	0.2674	0.2639	0.2713	0.2676	0.2710	0.2487	2.9

**Table 7:** Gradient elution procedures.

Time (t/min)	Acetonitrile (%)	0.2% Phosphoric Acid (%)
0	10	90
14	20	80
25	22	78
27	25	75
40	29	71
56	45	55
80	75	25
85	75	25

## Data Availability

Data are contained within the article. In addition, the data presented in this study are available on request from the corresponding author.

## Supplementary Materials

The following supporting information can be downloaded at: https://www.mdpi.com/article/10.3390/molecules29071517/s1, Figure S1: ^1^H NMR (500 MHz, CDCl_3_) spectrum of preconene II; Figure S2: ^13^C NMR (500 MHz, CDCl_3_) spectrum of preconene II.

## Abbreviations

VP: *Vernonia patula* Merr.; PC, *Praxelis clematidea*; AC, *Ageratum conyzoides* L.; AH, *Ageratum houstonianum* Mill; HPLC, high-performance liquid chromatography; CA, cluster analysis; PLS-DA, Partial Least Squares Discriminant Analysis.

## References

[1] Editorial Committee of the Flora of China, Chinese Academy of Sciences (1985). Flora of China.

[2] Guangxi Zhuang Autonomous Region Food and Drug Administration (2017). Quality Standards for Zhuang Medicine in Guangxi Zhuang Autonomous Region (Volume III).

[3] Liu Q., Yang J., Suo M. (2007). Study on the chemical composition and pharmacological activity of sesquiterpene lactones and steroidal saponins of vernonia. China J. Chin. Mater. Med..

[4] Wei Q., Xin C., Hui Z. (2018). Study on the chemical composition of *Vernonia patula*. J. Anhui Agric. Sci..

[5] Sun L., Ba Y., Yu L., Xu J. (2009). Progress in the research of pharmacological activities of the genus *Vernonia esulenta*. Xinjiang J. Tradit. Chin. Med..

[6] Wu P.S., Jeng J., Yang J.J., Kao V., Yen J.H., Wu M.J. (2020). *Vernonia patula (Dryand.)* Merr. and *Leucas chinensis (Retz.) R. Brown* exert anti-inflammatory activities and relieve oxidative stress via Nrf2 activation. J. Ethnopharmacol..

[7] Hira A., Dey S.K., Howlader M.S., Ahmed A., Hossain H., Jahan I.A. (2013). Anti-inflammatory and antioxidant activities of ethanolic extract of aerial parts of *Vernonia patula (Dryand.)* Merr. Asian Pac. J. Trop. Biomed..

[8] Zhou Y. (2012). Morphological characteristics of the invasive species Pseudo Stinky Grass and differences between similar species of Agastache rugosa. Guangdong For. Sci. Technol..

[9] Lin M., Kong D., Zhou L. (2020). Research progress on the biological characteristics and control of *Pseudomonas aeruginosa*. China Plant Prot..

[10] Cai Y., Zhu Y., Liu D., Teng J. (2006). Pharmacological identification of salted shrimp flowers. J. Chin. Med. Mater..

[11] Guo H., Liu J., Hu H., Wang X., Liu W. (2020). Determination of chlorogenic acid and caffeic acid content in the Zhuang Yao medicine *Vernonia patula* Merr. by HPLC. Hubei Agric. Sci..

[12] Liu W., Guo H., Qin J., Luo Y., Zheng L. (2021). Quality control of *Vernonia patula* Merr. based on multi-component content determination and principal component analysis. Chin. J. Hosp. Pharm..

[13] Zhang L., Huang Z., Lin Z., Zhong X., Cheng R., Huang Z. (2024). Microscopic identification characteristics and HPLC fingerprint analysis of *Patrinia villosa* and its counterfeit products. Chin. Tradit. Herbal. Drugs.

[14] Li H., Li F., Gao B., Guo J., Zhang Y., Jiang G., Yin X. (2024). A comparative study on the HPLC fingerprint and chemical pattern recognition of *Corydalis flavescens* and its related medicinal herbs. Chin. Tradit. Herbal. Drugs.

[15] Zheng C., Li W., Yao Y., Zhou Y. (2021). Quality evaluation of *Atractylodis macrocephalae rhizoma* based on combinative method of HPLC fingerprint, quantitative analysis of multi-components and chemical pattern recognition analysis. Molecules.

[16] Wang F., Qian Z., Liao G., Zeng J., Huang D., Liu Q., Xie X. (2022). HPLC coupled with chemical fingerprinting for multi-pattern recognition for identifying the authenticity of *Clematidis armandii Caulis*. J. Vis. Exp..

[17] Zhang H.J., Li H.R., Feng Z.Y., Li K., Hu Y.P., Feng S.X. (2020). Comparative study on HPLC fingerprints between crude and processed *Ligustri lucidi Fructus*. China J. Chin. Mater. Med..

[18] Wu L., Liang W., Chen W., Li S., Cui Y., Qi Q., Zhang L. (2017). Screening and analysis of the marker components in *Ganoderma lucidum* by HPLC and HPLC-MSn with the aid of chemometrics. Molecules.

[19] Liu M., Zhao X., Qiu Z., Sun L., Deng Y., Ren X., Mou J. (2022). Comparative investigation of the stems, leaves, flowers, and roots of *Centipeda minima* based on fingerprinting-multivariate classification techniques. J. AOAC Int..

