# Supplementary material for: Identification of Vernonia patula Merr. and Its Similar Varieties Based on a Combination of HPLC Fingerprinting and Chemical Pattern Recognition
Source: Molecules. 2024 Mar 28;29(7):1517. doi: 10.3390/molecules29071517 (PMC11013639; doi:10.3390/molecules29071517)
Supplement: Supplementary file 1 [file molecules-29-01517-s001.zip › molecules-2870466-supplementary.pdf]

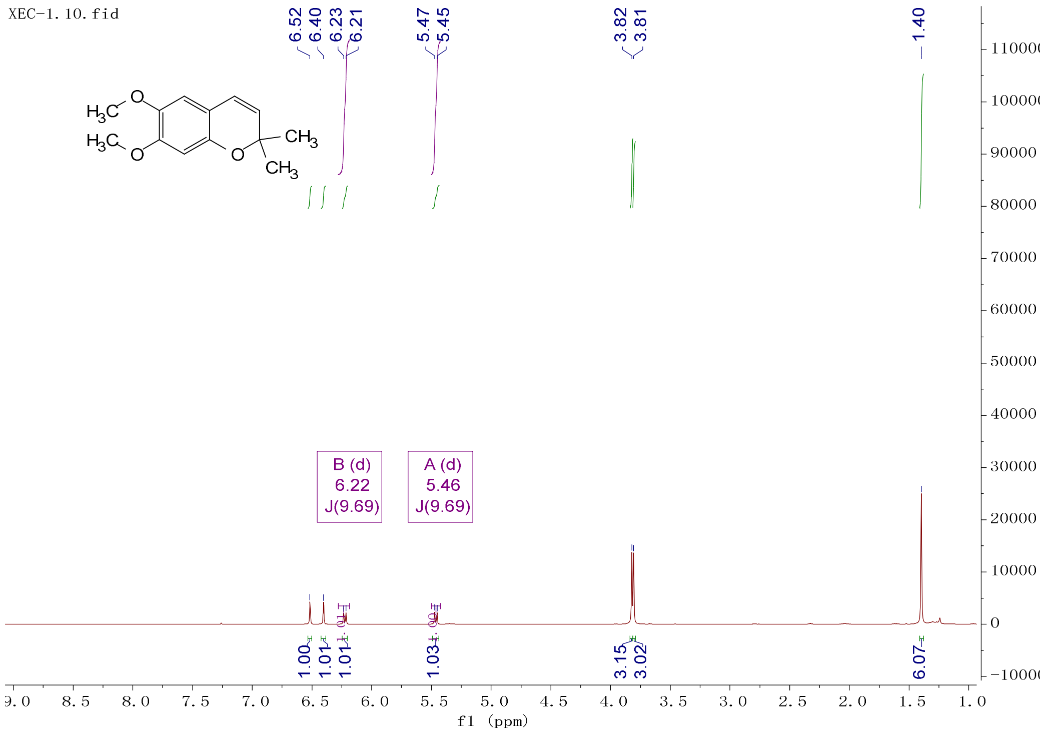

Figure S1. ^{1}H NMR (500 MHz, $CDCl_3$) spectrum of preconene II.

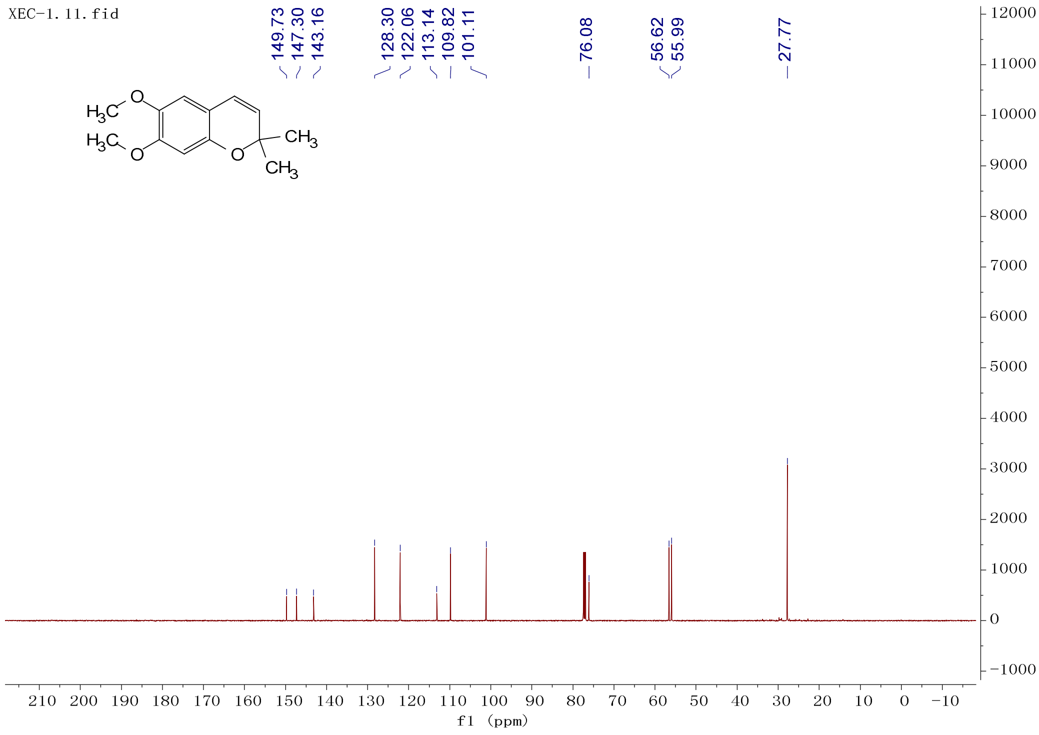

Figure S2. ^{13}C NMR (500 MHz, $CDCl_3$) spectrum of preconene II.
